# Supplementary material for: Rice Endosperm Protein Administration to Juvenile Mice Regulates Gut Microbiota and Suppresses the Development of High-Fat Diet-Induced Obesity and Related Disorders in Adulthood
Source: Nutrients. 2019 Dec 2;11(12):2919. doi: 10.3390/nu11122919 (PMC6950226; doi:10.3390/nu11122919)
Supplement: Supplementary file 1 [file nutrients-11-02919-s001.pdf]

SUPPLEMENTARY TABLES

Table S1, Compositions of the experimental diets

Ingredients (g/100 g)	Experimental diets					
	NFD-C	NFD-R	HFD-C	HFD-R	HFD-C-AA	HFD-R-AA
Casein	20.5		29.1			
REP		21.0		30.1		
Casein AAs					26.46	
REP AAs						26.46
Maltodextrin			6.0	6.0		
Corn starch	53.549	53.349	12.859	11.859	13.97	13.97
Sucrose	10.0	10.0	5.5	5.5	5.5	5.5
Soybean oil	6.2	5.9	2.0	2.0	2.0	2.0
Lard			33.0	33.0	34.0	34.0
Cellulose	5.0	5.0	6.6	6.6	6.6	6.6
Mineral mix*	3.5	3.5	3.5	3.5	3.5	3.5
Vitamin mix†	1.0	1.0	1.0	1.0	1.0	1.0
Calcium carbonate			0.18	0.18		
Choline bitartrate	0.25	0.25	0.25	0.25	0.25	0.25
Tert-butylhydroquinone	0.0014	0.0014	0.0014	0.0014	0.0014	0.0014

NFD-C, normal-fat diet (NFD) with casein as the protein source; NFD-R, NFD with rice endosperm protein (REP) as the protein source; HFD-C, high-fat diet (HFD) with casein as the protein source; HFD-R, HFD with REP as the protein source; HFD-C-AA, HFD-C with amino acid (AA) mixtures simulating the AA compositions of casein; and HFD-R-AA, HFD-R with AAs simulating the AA compositions of REP. *AIN-93G mineral mix (Oriental Yeast Co., Ltd., Tokyo, Japan). †AIN-93G vitamin mix (Oriental Yeast Co., Ltd.).

Table S2, Amino acid compositions of casein and REP

Amino acid compositions (μg/mg protein)	Casein	REP
Asp	73.0	95.9
Thr	43.0	35.8
Ser	54.0	51.8
Glu	220.0	179.6
Gly	19.0	44.7
Ala	31.0	54.9
Val	70.0	61.1
Cys	5.0	10.5
Met	31.0	19.0
Ilu	56.0	43.5
Leu	97.0	85.8
Tyr	57.0	55.6
Phe	53.0	58.1
Lys	83.0	34.9
His	31.0	29.5
Arg	38.0	90.3
Pro	120.0	44.8
Trp	12.0	13.0
Total	1093.0	1008.8

Data on the amino acid compositions of casein were provided by Oriental Yeast Co., Ltd. (Tokyo, Japan), whereas those of REP were reported by Kumagai et al. (Supplementary Reference 1).

Table S3, Metabolic rates in the four groups and the effects of diet on each parameter

Parameters	Groups				*P* values		
	CC	RC	CR	RR	Juvenile-period protein source	Adulthood protein source	Interaction
VO_2 (mL/kg/h)	2985.4 ± 272.8	3057.5 ± 204.6	2916.3 ± 246.6	3054.6 ± 314.1	0.213	0.667	0.693
RER	0.83 ± 0.19	0.84 ± 0.21	0.88 ± 0.28	0.82 ± 0.17	0.714	0.903	0.610
Energy consumption (Kcal/h)	0.55 ± 0.11	0.54 ± 0.08	0.54 ± 0.11	0.53 ± 0.11	0.649	0.784	1.000

RER, respiratory exchange ratio. Data are shown as means ± standard deviation.

Table S4, Fatty acid and bile acid excretion in the feces of the four groups and the effects of diet on each parameter

Parameters	Groups				*P* values		
	CC	RC	CR	RR	Juvenile-period protein source	Adulthood protein source	Interaction
Total lipids (mg/day)	12.13 ± 1.37	14.04 ± 1.35	14.94 ± 1.61	14.69 ± 1.50	0.081	<0.001	0.025
Palmitic acid (mg/day)	1.09 ± 0.25	1.55 ± 0.27	1.54 ± 0.19	2.03 ± 0.58	<0.001	<0.001	0.913
Stearic acid (mg/day)	0.80 ± 0.17	0.94 ± 0.14	1.26 ± 0.20	1.23 ± 0.28	0.415	<0.001	0.208
Total saturated fatty acid (mg/day)	1.94 ± 0.42	2.52 ± 0.34	2.87 ± 0.38	3.31 ± 0.71	0.002	<0.001	0.653
Oleic acid (mg/day)	0.97 ± 0.34	0.96 ± 0.22	1.07 ± 0.22	1.38 ± 0.41	0.133	0.011	0.098
Linoleic acid (mg/day)	0.14 ± 0.03	0.15 ± 0.03	0.18 ± 0.03	0.17 ± 0.07	0.883	0.023	0.825
Total unsaturated fatty acid (mg/day)	1.12 ± 0.37	1.10 ± 0.25	1.25 ± 0.24	1.56 ± 0.42	0.164	0.008	0.127
Total bile acids (mg/g feces)	1.34 ± 0.28	1.87 ± 0.50	1.75 ± 0.47	2.52 ± 0.43	<0.001	<0.001	0.396

Data are shown as means ± standard deviation.

Table S5, Correlations between the relative abundances of gut microbiota genera and study parameters

Taxa	Relative abundances in the four groups				Two-way ANOVA *P*-values				Correlation coefficient *P*-values											
	CC	RC	CR	RR	Juvenile - period protein source	Adulthood protein source	Interaction	Tukey (CC vs. RC)	Body weight	HOMA -IR	UAE	Kidney weight	Liver weight	Vacuolar area	Mesangial area	Glomerular area	LBP	Serum TNF-α	Kidney TNF-α	Liver TNF-α
g_*Lactobacillus*	0.023	0.042	0.016	0.076	0.006	0.317	0.140		0.145	0.322	0.029	0.075	0.354	0.076	0.024	0.175	0.058	0.329	0.753	0.079
c_Clostridia; Other; Other; *Other*	0.004	0.008	0.005	0.003	0.255	0.022	0.003	0.015	0.796	0.062	0.530	0.680	0.841	0.567	0.918	0.877	0.830	0.885	0.327	0.479
f_Christensenellaceae	2E-04	1E-04	9E-05	4E-05	0.013	<0.001	1.000		0.091	0.640	0.202	0.175	0.250	0.013	0.032	0.088	0.406	0.857	0.625	0.777
f_Clostridiaceae; *Other*	0.007	0.011	0.002	0.002	0.009	<0.001	0.004	0.005	0.076	0.189	0.010	0.891	0.558	0.206	0.024	0.190	0.263	0.193	0.154	0.452
g_*Dehalobacterium*	0.002	0.002	0.001	0.001	0.023	<0.001	0.188		0.008	0.142	0.011	0.277	0.007	0.237	0.024	0.059	0.154	0.032	0.465	0.074
f_Lachnospiraceae	0.039	0.014	0.039	0.044	0.012	<0.001	<0.001	0.007	0.887	0.136	0.061	0.232	0.764	0.752	0.714	0.516	0.814	0.047	0.993	0.129
f_Lachnospiraceae; g_*Ruminococcus*	0.039	0.031	0.048	0.028	0.001	0.468	0.158		0.436	0.380	0.220	0.010	0.219	0.236	0.177	0.471	0.624	0.093	0.590	0.307
g_*Allobaculum*	0.230	0.162	0.132	0.075	0.001	<0.001	0.759		<0.001	0.476	0.001	0.069	0.026	<0.001	<0.001	<0.001	<0.001	0.413	0.097	0.082
g_*Escherichia*	0.008	0.001	4E-04	0.002	0.002	<0.001	<0.001	0.001	0.006	0.013	0.002	0.042	0.042	<0.001	<0.001	0.001	0.030	0.072	0.012	0.024

HOMA-IR, homeostasis model assessment of insulin resistance; UAE, urinary albumin excretion; LBP, lipopolysaccharide-binding protein; TNF-α, tumor necrosis factor-α.

Supplementary References

1 Kumagai, T. *et al.* Production of rice protein by alkaline extraction improves its digestibility. *J Nutr Sci Vitaminol (Tokyo)* **52**, 467-472 (2006).
